# LLM-Based Unknown Function Automated Modeling in Sensor-Driven Systems for Multi-Language Software Security Verification

**DOI:** 10.3390/s25092683

**Published:** 2025-04-24

**Authors:** Liangjun Deng, Qi Zhong, Jingcheng Song, Hang Lei, Wenjuan Li

**Affiliations:** 1 School of Information and Software Engineering, University of Electronic Science and Technology of China, Chengdu 610054, China; dengliangjun@skycto.com (L.D.); hlei@uestc.edu.cn (H.L.); 2 Faculty of Data Science, City University of Macau, Macau, China; 3 School of Information Science and Engineering, Linyi University, Linyi 276000, China; sjc191500132@gmail.com; 4 Department of Mathematics and Information Technology, The Education University of Hong Kong, Hong Kong, China; lwenjuan@eduhk.hk

**Keywords:** WebAssembly, vulnerability verification, symbolic execution, LLM, sensors

## Abstract

The rapid expansion of the Internet of Things (IoT) has made software security and reliability a critical concern. With multi-language programs running on edge computing, embedded systems, and sensors, each connected device represents a potential attack vector, threatening data integrity and privacy. Symbolic execution is a key technique for automated vulnerability detection. However, unknown function interfaces, such as sensor interactions, limit traditional concrete or concolic execution due to uncertain function returns and missing symbolic expressions. Compared with system simulation, the traditional method is to construct an interface abstraction layer for the symbolic execution engine to reduce the cost of simulation. Nevertheless, the disadvantage of this solution is that the manual modeling of these functions is very inefficient and requires professional developers to spend hundreds of hours. In order to improve efficiency, we propose an LLM-based automated approach for modeling unknown functions. By fine-tuning a 20-billion-parameter language model, it automatically generates function models based on annotations and function names. Our method improves symbolic execution efficiency, reducing reliance on manual modeling, which is a limitation of existing frameworks like KLEE. Experimental results primarily focus on comparing the usability, accuracy, and efficiency of LLM-generated models with human-written ones. Our approach was integrated into one verification platform project and applied to the verification of smart contracts with distributed edge computing characteristics. The application of this method directly reduces the manual modeling effort from a month to just a few minutes. This provides a foundational validation of our method’s feasibility, particularly in reducing modeling time while maintaining quality. This work is the first to integrate LLMs into formal verification, offering a scalable and automated verification solution for sensor-driven software, blockchain smart contracts, and WebAssembly systems, expanding the scope of secure IoT development.

## 1. Introduction

With the rapid development of the Internet of Things (IoT) [1,2], software defects not only threaten financial stability and reputation but also pose severe risks to human safety. This is especially so in sensor-driven systems, where real-time decision-making is paramount. Ensuring software correctness and identifying vulnerabilities are critical in modern software development [3]. Symbolic execution is a key technique for software security verification, capable of detecting potential vulnerabilities and providing mathematical proof of correctness in formal verification. This is particularly essential in safety-critical and mission-critical systems, such as industrial automation [4], autonomous vehicles [5], etc., where software failures can lead to catastrophic consequences [6]. As sensor networks become more integral to intelligent systems, the role of symbolic execution in software validation and security assurance [7,8] continues to expand, ensuring that software interacting with real-world data remains robust, reliable, and secure.

However, symbolic execution still faces many challenges [9], with unknown function modeling being one of the most crucial problems. We have identified the following primary scenarios for this issue: (1) The absence of an executable environment where certain device APIs used in production cannot be simulated in the testing environment. (2) The difficulty in symbolic tracing due to memory isolation caused by network operations (e.g., web services and database connections) that disrupt symbol tracking. (3) Reliance on third-party and system libraries when the source code is unavailable, leading to unpredictable outcomes from library functions, which hinders accurate modeling. We define “unknown functions” as APIs without accessible source codes or those with indeterminable outcomes.

Predictably, symbolic execution is becoming increasingly constrained in the context of modern software. The development of IoT and edge computing has shifted the focus of traditional software security detection. Modern software systems are now integrated across diverse environments, such as autonomous vehicles, medical systems, and Web3.0 applications. For instance, Jodogne [10] used WebAssembly to render medical images, bridging the gap between web-based and desktop-based medical applications. Traditional security verification faces three primary challenges, namely, the emergence of a new assembly language program, a new runtime system environment, and the server communication interface in the embedded system. To address the issues, the response data from a system environment or a server interface are necessary. However, rebuilding a system in a real environment for testing is highly inefficient because certain inputs are difficult to implement. Therefore, the emulator serves as a practical alternative.

In vulnerability verification, two general methods are employed for emulation, namely, system simulation, which reproduces the exact interface and behaviors of the original system, and lightweight function simulation, which simulates interface behavior without replicating the entire system, offering a more streamlined solution. In general, function simulation is widely favored due to its efficiency and simplicity. Considering the complexity of application program interfaces (APIs) in the real world, traditional algorithms categorize them, build frameworks, and manually develop functional code to simulate various interface behaviors as needed. This highlights the significant manual effort required for unknown function modeling, which depends heavily on the skill and efficiency of the developer.

To reduce this dependency, this paper proposes an automated approach based on machine learning to generate models for unknown functions. Our method minimizes human intervention by fine-tuning an autoregressive language model with 20 billion parameters while preserving the core principles of symbolic execution. The contributions of this paper are as follows:

- Automated coding for vulnerability verification: Traditional unknown function modeling frameworks require extensive manual coding. Our approach leverages the automated coding capabilities of artificial intelligence to replace human effort, significantly improving the efficiency of vulnerability verification.
- Reasoning with LLMs for formal verification: Unknown functions span various categories, including external system functions, network interfaces, and hardware interfaces. Traditional frameworks rely on manual information collection to model APIs. This paper is the first to utilize the reasoning capabilities of LLMs in formal verification tasks. By enhancing automation in software security verification, this work offers a novel perspective on integrating machine learning to address the challenges of modeling frameworks. It effectively resolves numerous issues previously encountered in real-world symbolic execution scenarios involving unknown functions.
- Multi-language program support: Multilingual program analysis is a critical challenge, as traditional symbolic execution engines typically support only a single programming language. Our research explores the combination of WebAssembly with symbolic execution, providing a new research reference point for multi-language program vulnerability verification. This includes support for languages such as C/C++, Rust, Golang, etc., improving the feasibility of symbolic execution in the industrial applications of modern software.

The rest of this paper is arranged as follows. Section 2 reviews the related works. Section 3 introduces the preliminaries, while Section 4 provides an extensive discussion of the solutions developed to address the challenges encountered during the experimental phase. Section 5 meticulously documents the experimental process and presents the outcomes associated with automating the modeling of unknown functions within symbolic execution. Section 6 concisely summarizes the significance of this work, providing a valuable reference for integrating LLMs into symbolic execution to improve the automation of vulnerability verification.

## 2. Related Works

The first approach to handling unknown functions in symbolic execution is concrete execution, where APIs are called to directly generate results. The combination of concrete results and original symbols is referred to as ‘concolic’ execution [11,12]. Although this approach partially mitigates the challenges associated with unknown functions, it may still fail to capture certain logical execution paths due to the inherently unpredictable behavior of such functions. Specifically, while it addresses the issue of executing unknown functions, the concolic (concrete + symbolic) execution technique does not resolve the problem of modeling their internal behavior. That is, the return values of these functions cannot be symbolically traced or fully represented, resulting in incomplete symbolic information and potentially leading to missed execution paths. An advanced variant of this approach is fuzz testing, which operates by executing concrete, randomly generated inputs within a fully simulated environment, as opposed to relying on symbolic inputs. However, a significant limitation of fuzz testing lies in its inability to achieve comprehensive code coverage [13], primarily due to the inherent randomness in input generation, as opposed to a principled modeling of program behavior. Alternatively, functional modeling offers a potential solution by approximating the behavior of unknown functions. Nevertheless, this method typically requires manual intervention, thereby constraining the extent to which verification processes can be performed intelligently and autonomously. The neural network model has also been a hot scheme in recent years. Previous deep learning research concentrated on identifying vulnerable code features or logical flow, but often struggled to precisely pinpoint defects. Furthermore, both true negatives and false positives remain inevitable, and there are no obvious advantages over traditional methods. On the other hand, with regard to functional modeling proposed by traditional methods, KLEE designed an API framework that isolates real interfaces [14,15]. While similar to our work, KLEE’s approach suggests implementing various strategies tailored to runtime environment requirements. This typically necessitates significant developer involvement, as the framework relies on manually written code to handle all possible responses of an unknown function. In contrast, our work also employs an isolation framework to simulate real API responses but replaces manual coding with AI-based automated function modeling. While neural networks are inherently unexplainable, symbolic execution offers a mathematically explainable proof mechanism. By integrating LLMs with traditional verification frameworks, our approach enhances the efficiency of conventional tools, offering a more automated and robust solution. In addition, we integrate this approach into one of our previous work projects on formal validation as a platform service (FVPS) [16], slowing down the manual modeling effort and increasing the level of automation across the platform.

## 3. Preliminaries

### 3.1. WebAssembly

WebAssembly is a rapidly evolving and highly promising technology that forms the foundational support for our experimental research. It solves the problem of cross-language compatibility, supporting languages such as C 99, C++ 98, Rust 1.x, Golang 1.11, Python 3.x, and C# .Net 5. In addition, its instruction characteristics are particularly well-suited for symbolic execution analysis. Moreover, our research contributes to enhancing the security of WebAssembly programs. WebAssembly [6,17] is an efficient and lightweight instruction set that perfectly supports all types of central processing units (CPUs). As an official standard of the World Wide Web Consortium (W3C), it represents the next generation of instruction sets. The WebAssembly core specification defines the semantics of WebAssembly modules independently of any specific embedding, ensuring its portability and flexibility [18].

WebAssembly’s fast startup, high-performance execution, and cross-CPU architecture make it a superior containerization or sandbox technology. It has been widely adopted across diverse domains, including blockchain [19], cryptography, simulation, deep learning, healthcare [20], and embedded microcontrollers, demonstrating strong performance outcomes. Haas [21] noted that WebAssembly has significantly enhanced the capabilities of the web, while Lehmann [22] argued that WebAssembly is reviving numerous concepts, including binary security and program analysis [23,24], as well as applications in edge computing, containerization, microservices, IoT [25], and serverless architectures. Hilbig [23] advocated for empirical studies focused on the security, languages, and real-world use cases of WebAssembly binaries. The program analysis of WebAssembly presents many challenges for researchers, particularly in terms of understanding its security and optimizing its use in diverse applications.

Watt et al. [26] demonstrated the effectiveness of using Isabelle/HOL and Coq with interactive theorem proving to identify defects in two specifications of Wasm 1.0. They also implemented WasmCert-Isabelle and WasmCert-Coq, based on WebAssembly. Octopus is a Python-based security analysis framework designed for Wasm modules, utilizing Microsoft’s Z3-solver for tasks like disassembly and control flow analysis [27]. While Octopus is a valuable tool for understanding Wasm bytecode and detecting defects, it cannot independently perform symbolic execution on modules, limiting its use to supporting control flow defect detection research. At present, there are few applications of machine learning in formal verification. Among them, ContractWard [28] suggests using machine learning techniques to detect vulnerabilities in smart contracts. While effective for known security issues, this approach struggles to adapt to emerging security risks. Similarly, other machine learning research [29] has been applied to vulnerability verification tools but remains limited in scope. In contrast, we are the first to propose the use of LLMs for modeling unknown functions in the context of security verification. We apply this innovative approach to WebAssembly and demonstrate its effectiveness in modeling and verification. This contribution marks a significant advancement in the field of formal verification and promises to have a positive impact on software engineering.

### 3.2. Large Language Model

LLMs such as GPT-3, GPT-4, and GitHub copilot 3.10 have revolutionized code generation, enabling the automation of coding tasks through natural language inputs. These models are capable of generating code, offering coding suggestions, automating code completion, fixing bugs, and suggesting best practices, significantly enhancing programming speed and efficiency. For instance, GitHub Copilot [30] provides real-time coding suggestions, and tools like ChatGPT 4o have even been used by students for college-level coding assignments [31]. AlphaCode [32] attempts to generate competition-level programming codes. Castelvecchi [33] raised the question of whether tools like ChatGPT and AlphaCode could eventually replace programmers. Building on the findings of these studies, we intend to fine-tune a generative LLM, specifically GPT-NeoX-20B, to assist in the automated modeling of unknown functions within symbolic execution.

GPT-NeoX-20B is a 20-billion-parameter autoregressive language model trained on the Pile [34]. It is based on NVIDIA’s Megatron language model and enhanced with techniques from DeepSpeed [35]. It is in widespread use in academia, industry, and government labs [36]. Unlike specialized models like ChatGPT, which have been optimized for tasks such as writing and chatting through techniques like reinforcement learning from human feedback (RLHF), GPT-NeoX-20B has not been tailored for specific tasks. To use it effectively for symbolic execution and modeling unknown functions, as discussed in this paper, it needs to be fine-tuned for these particular tasks.

### 3.3. Symbolic Execution

Symbolic execution, as surveyed by Baldoni et al. [37], systematically explores all potential paths a program may take, achieving comprehensive code coverage and identifying rare edge cases that are often overlooked by standard testing methods. Unlike fuzzy or black-box testing, which may redundantly test the same path, symbolic execution assesses each logical path once, ensuring precision and eliminating false positives. By using symbolic variables instead of concrete values to represent program operations, symbolic execution translates the program into a set of mathematical formulas, enabling the automatic generation of test cases that uncover vulnerabilities and errors. This process streamlines debugging and enhances code quality. Ideally, symbolic execution can explore all program paths and detect vulnerabilities.

However, symbolic execution often struggles with analyzing external library interactions and system calls, leading to partial analysis. A significant limitation involves the handling of unknown functions, which has traditionally limited its performance and applicability in real-world scenarios. The introduction of concolic execution offered a practical solution by trading off some coverage for industrial usage. Concolic execution continues the symbolic analysis using concrete inputs instead of symbolic ones. APIs must return sufficient status values for their functionality; otherwise, this compromises the logical coverage of symbolic execution. A common approach to address this issue is to create abstract function models that return appropriate values for upper-level calls, rather than relying on actual API calls. However, the manual modeling of unknown functions by developers presents challenges related to manual intervention and the scalability of the models.

### 3.4. Blockchain

Blockchain technology has demonstrated substantial potential across a wide spectrum of applications within the Internet of Things (IoT), primarily through its capacity to ensure the security, immutability, and auditability of sensor data—attributes that are fundamental to the integrity and reliability of IoT ecosystems. Among various blockchain frameworks, Hyperledger Fabric has emerged as a particularly suitable solution for integration with IoT environments. It facilitates the protection and verification of critical sensor data, such as environmental metrics and industrial telemetry, rendering such data tamper-resistant and verifiable. This capability supports secure, decentralized, and automated decision-making within intelligent IoT systems. Despite the transformative potential of blockchain-based smart contracts in enabling trustless and automated transactions, they remain vulnerable to a range of security threats. These vulnerabilities are intensified by the inherent complexity of blockchain infrastructures and the challenges associated with their accurate simulation. Emulating real-world blockchain conditions proves to be highly non-trivial. Existing testing frameworks often fall short in replicating adversarial behaviors or rare edge-case scenarios. Moreover, current tools exhibit limitations in modeling complex inter-contract interactions, cross-chain dependencies, and multi-layered protocol structures. Among them, FVPS [16] implements an abstract function layer of the blockchain running environment based on WebAssembly to automate the detection of smart contracts. The downside of this approach is that it requires manual implementation of the abstract function model. Our proposed automated modeling approach will extend this project’s ability to automate the validation of systems in complex network environments.

## 4. Methodology

In this section, we present the entire implementation process, which encompasses numerous detailed steps. The key steps to be aware of in this process are explained in this section. First, let us take a look at the framework as a whole. The whole process is divided into data preparation, model training, and model application. In the data preparation phase, our goal is to obtain the Q&A corpus, and the simplest input is a function definition as the human part of the human–bot Q&A pair. The ‘bot’ outputs Python implementation codes for functions. In order to generate the corpus of the ‘human’ part, we first need to collect the API categories in Table 1. We use prompt text to enable LLMs to simulate the role of an interviewed programmer, following the prompt to implement the function. The LLM answers the code implementations. In fact, the preparation is a very complex process, and most of the data are synthesized by machines. For example, the official WebAssembly organization provides interface specifications and definitions for clarity. We synthesized a human–bot question–answering pair corpus. In this preparation process, we also make use of common LLMs to assist us in our work. In the model training phase, GPT-NeoX is modified to fit our hardware. Finally, when the model was applied, it was embedded in one of our previous works to verify the feasibility of the method. A detailed description will be given in a later section.

**Figure 1:**
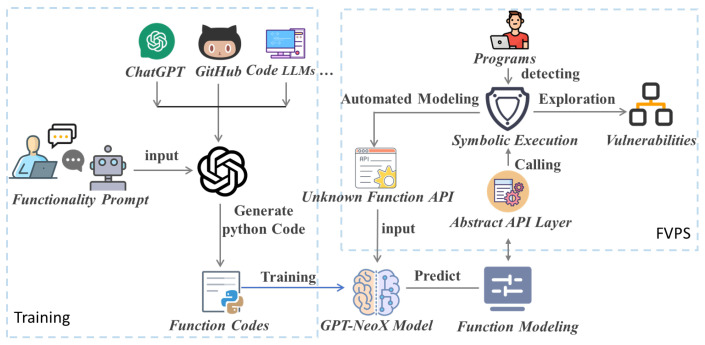
The system processing of automated unknown function modeling in symbolic execution. It shows the synthesis process of training a corpus from public LLMs, a large amount of Python code is synthesized based on question–answering templates for training a large-scale language model. The symbolic execution engine inputs an unknown function API declaration from the abstract API layer, while the LLM outputs the function implementation in Python. This ensures that the external functions have specific returns during symbolic execution, allowing state exploration of symbolic execution to continue.

We mainly use a pre-training approach to achieve the automatic modeling of unknown functions based on LLMs. To realize the automatic modeling of unknown functions within symbolic execution, our research methods involve several key components: the training and fine-tuning of a 20B LLM, optimization of GPT-NeoX, construction of an abstract layer for WebAssembly functions, and the application to blockchain smart contracts. Among them, the symbolic execution framework, including the unknown function API, the abstract API layer, path exploration, vulnerabilities, etc., is based on our previous research work FVPS [16]. Function modeling replaces earlier manual approaches. Our processing flow is shown in Figure 1 below.

Build training datasets: The first step in automating the modeling of unknown functions is to collect as many common APIs as possible from runtime environments. These APIs span various categories, including programming languages, standard libraries, third-party libraries, and business logic code. Programming languages typically have language-specific grammatical features. For instance, the ‘print’ function outputs formatted text to the console, but the exact function name varies across languages, such as the ‘Println’ function of the ‘fmt’ package in Golang, ‘cout’ in C++, and ‘println’ in Rust. Although these functions serve similar purposes, they differ in their implementation for symbolic execution.

When compiling WebAssembly using different compilers, external runtime standard libraries are introduced, such as LLVM 8, Golang 1.11, Rust 1.x, Emscripten 1.37, Clang 9.0, and Node.js 12.x. Each library has a unique API, and while they may appear as syntactic sugar, symbolic execution must carefully consider the implementation of each API. Another example is the WebAssembly system interface (WASI) standard library, which is a set of APIs developed by a subgroup of the WebAssembly Community Group. While these APIs serve similar purposes across different runtime environments (e.g., Linux, browsers), they may have different names. In real-world program development, developers often depend on some base frameworks, such as those found in blockchain smart contracts (e.g., Hyperledger). The Hyperledger libraries contain framework-specific APIs like ‘stub.GetFunctionAndParameters’, which are unknown to symbolic execution. System APIs such as ‘gettime’, ‘getdate’, ‘I/O stream’, ‘thread’, ‘getip’, ‘ioctl’, and ‘fork’ are also common in symbolic execution. We collect common APIs across a wide range of use cases, including text and file handling, configuration files, image reading and writing, threading, operating systems, sockets, HTTP, RESTful, web services, logs, databases, encryption, and user input, as summarized in Table 1.

**Figure 2:**
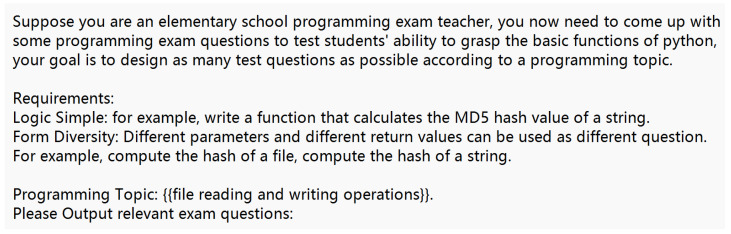
A prompt template for coding task generation according to a program topic.

To model unknown functions effectively, we design corresponding prompt templates to generate Python function codes using LLMs. The main goal of this process is to create comprehensive function models that account for exceptions, special values, random values, etc., rather than focusing on the actual implementation of a function. For example, when modeling an encryption function, it is critical to consider special return values that might be used in concrete conditions. For code tasks, collecting data manually is too costly. To replace manual mechanisms, obtaining datasets through feedback mechanisms executed by LLMs is our primary approach. Firstly, Figure 2 shows one of our prompt templates for coding task generation according to a program topic from Table 1. Secondly, part of the coding task problem is supplemented manually, such as third-party libraries. Building upon this foundation, we utilize LLMs to rephrase synonyms, thereby expanding the pool of questions.

**Figure 3:**
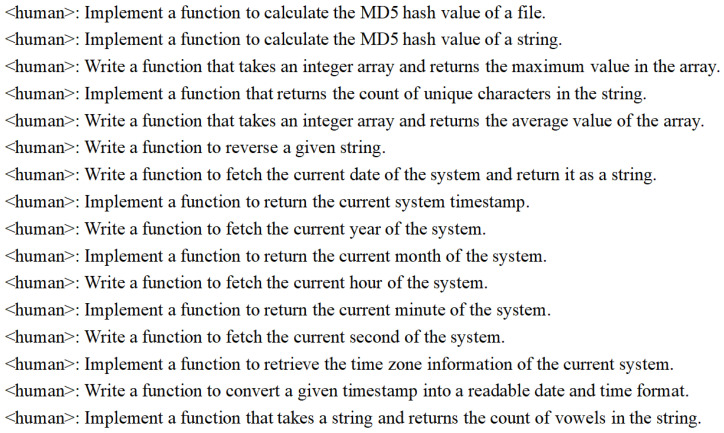
Basic coding tasks generated by ChatGPT using prompt templates, based on Table 1.

Unknown function modeling prioritizes capturing the full range of potential return values, distinguishing it from standard function implementation. We employ LLMs to simulate the role of an interviewer using template questions, extending these functions to include exception handling, error resolution, performance optimization, coding parameters, and more. This approach provides a more complex function return model. The same logical requirements create many coding tasks with variations in parameter forms or return forms. A portion of these coding tasks is illustrated in Figure 3.

In order to build a rich and flexible training dataset, the next step involves generating concrete Python codes from these coding tasks. Since code implementations tend to be fixed, we parameterize the generated function code into templates. This parameterization enables the GPT-NeoX model to learn the underlying patterns across a diverse set of codes. For instance, consider the HTTP status response from a server via a POST API. Under normal conditions, the server returns a 200 status code, as shown in Equation (1):

(1) $$f\left(x_{i}\right)=200\;\mathrm{for}\;x_{i}\in \left\{x_{1},x_{2},x_{3},\ldots \right\},$$

here, $\left\{x_{1},x_{2},x_{n},\ldots \right\}$ denotes various possible situations. However, status codes such as 404, 503, and others must also be returned under specific conditions, each with a defined probability. Therefore, the ‘POST’ function generates additional function codes to model these status codes based on different odds. Equation (2) illustrates the parameter generalization formula.

(2) $$f\left(x\right)=\left\{\begin{array}{c} 200\;\;\mathrm{if}\;x\;\mathrm{is}\;\mathrm{valid}\;\mathrm{request}\\ 404\;\;\mathrm{if}\;x\;\mathrm{is}\;\mathrm{invalid}\;\mathrm{URL}\\ 500\;\;\mathrm{if}\;x\;\mathrm{triggers}\;\mathrm{server}\;\mathrm{error}\\ \cdots \end{array}\right.$$

**Figure 4:**
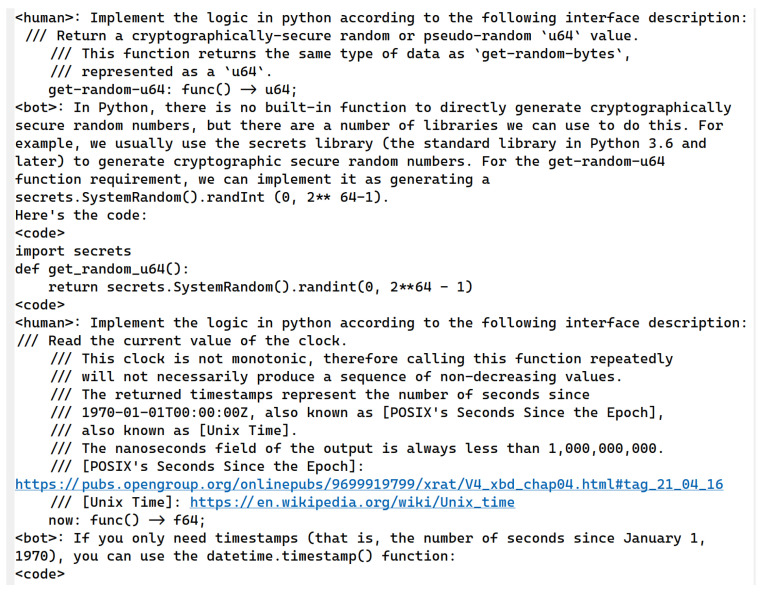
Trained corpus automatically generated by coding LLMs via question templates. The symbols ‘**’ in this picture represent powers in the Python language.

The coding tasks in Figure 3 generate code implementations. After parameter generalization, as shown in Equation (2), we create a variety of training corpora. By removing duplicates, we compile a total of 50,000 original questions. These questions were then processed using the coding LLMs, which generates answers, yielding approximately 45,000 data items after filtering. To enhance the dataset, we employ synonyms to expand the variety of function names, enabling the model to adapt to various environmental contexts during function modeling. Examples include variations such as ‘fd_write’ in LLVM v8, ‘shim.Error/shim.Success’ in Hyperledger 1.4, and ‘console.log/console.debug/console.info’ in JavaScript ES5. The correspondence between function declarations and their respective implementations forms the basis of our training corpus. In addition, the WebAssembly official organization provides interface specifications and definitions, clarifying that developers are responsible for specific implementations. Hence, coding LLMs are used to generate the implementations as a training corpus, as shown in Figure 4.

In conclusion, we prepared four different levels of training instruction templates to generate the training datasets. In general, the function declarations of system APIs and common SDKs and their APIs already contain rich function information to build functional logic code, although there are differences in different operating systems and programming languages. Through experiments, it is shown that such differences can be fully understood by the GPT-NeoX model. Therefore, our experiment prepares the end-to-end training corpus according to the declaration of the functional model to reduce the workload of manual intervention as much as possible. The second training instruction template is based on function description and function declaration. Most unknown functions are difficult to process uniformly, mainly because the data exchange and communication interfaces between information systems are self-defined. This part of the self-defined API usually has interface documents describing the input and output of related API interfaces. The third training instruction template is based on the user’s special requirements and function declarations. To obtain the system time, for example, if there are no special requirements, the model will output the current time of the system. If the software function is to determine the past or future time, then the current time of the system cannot trigger the corresponding logical conditions, resulting in incomplete coverage of software vulnerability detection. So specific requirements are added to the function declaration to achieve the goal, such as the system time returning a certain range of values. The fourth instruction training template combines requirements, comments, and declarations. The training instruction templates are shown in Table 2.

**Figure 5:**
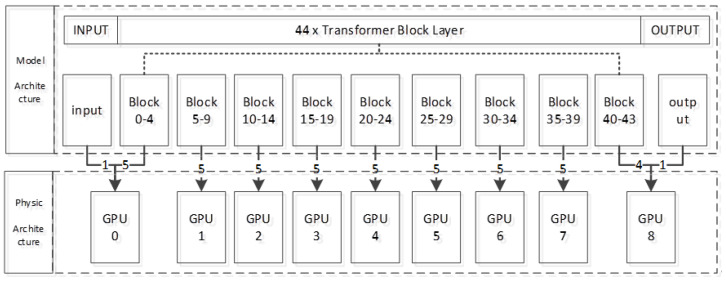
A 20–billion–parameter autoregressive language model, involving 44 Transformer decoder block layers, is distributed over 9 GPUs with 48 gigabytes of memory.

Pretrained model: GPT-NeoX-20B serves as the base model, a simple question–answer prompt template for constructing the unknown function model is designed to train the LLM. The original trained architecture of GPT-NeoX-20B requires the resources of 96 GPUs, each equipped with 80 gigabytes of memory. Due to hardware constraints, the existing equipment cannot support the continuous training of this model. Our study reduces the training architecture to 9 GPUs with 48 gigabytes of memory. Through pipeline parallelism, the 44 Transformer block layers of the pre-trained model are evenly split into 9 GPUs, and each GPU loads 5 block layers. Since 44/9 cannot be calculated as an integer, the ninth GPU8 only loads the last four block layers of the model, and the input layer and output layer are loaded to the beginning GPU0 and the last GPU8, respectively, to construct the topology of the entire trained model, as shown in Figure 5.

**Figure 6:**
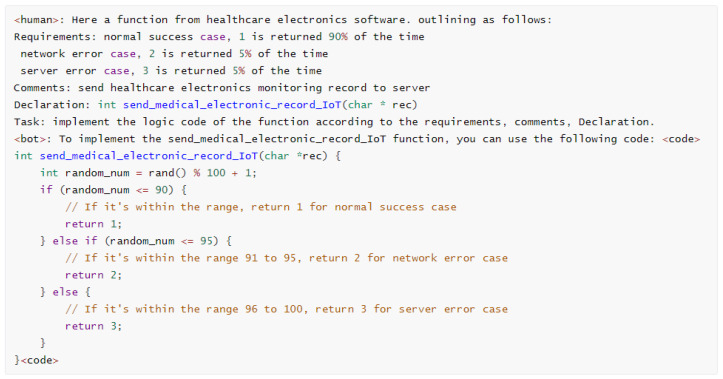
A prompt template for a large language model’s downstream task generates function logic code corresponding to the declarations, comments, and special requirements of a function. The logic code is wrapped by a pair of special tokens ‘<code>’. The symbol ‘*’ in this picture denotes a pointer of char.

The prompt template consists of the bootstrap, modeling requirements, function comments, function declarations, and question and answer, as shown in Figure 6. In the prompt template, the API declaration section is the most important and mandatory; this is the minimum requirement for automation. The requirement section is from the content reference enumeration or the API interface description document. The other sections consist of fixed words to bootstrap NLP inferences. Consequently, in order to extract Python codes from LLM responses, the response source code is enclosed within the special token ‘<code>’.

Transformer: In order to implicitly learn the positional information of sequences. The Transformer network suggests a positional encoding (PE) methodology, called the 2D PE tensor, as shown in Equation (3):

(3) $$\left\{\begin{array}{c} PE_{\left(pos,2i\right)}=\sin\left(pos/10000^{2i/d}\right),\\ PE_{pos,2i+1}=\cos\left(pos/10000^{2i/d}\right). \end{array}\right.$$

This positional method combines the position index and dimension index, leading to each tensor element’s position independence. ‘pos’ denotes the position of the word in the sentence, ‘*d*’ denotes the dimension of PE, ‘2i’ denotes the even dimension, and ‘2i+1’ denotes the odd dimension (2i≤d,2i+1≤d). This position encoding method can adapt to sentences that are longer than all the sentences in the training sets. Additionally, for computational convenience, absolute positional encoding is employed, meaning that each position in the sequence is assigned a fixed position vector. The final input representation of each word is derived by combining its word vector and position vector, while the dense matrix is obtained through the compression of the embedding matrix.

(4) $$A_{i,j}^{abs}={\left(W_{q}\left(E_{x_{i}}+U_{i}\right)\right)}^{T}\left(W_{k}\left(E_{x_{j}}+U_{j}\right)\right).$$

The attention score calculation in the Transformer network is shown in Equation (4). $E_{x_{i}}$ and $E_{x_{j}}$ are word embeddings for $x_{i}$ and $x_{j}$, and $P_{i}$ and $P_{j}$ are the position vectors for positions i and j. When $W_{q}$ and $W_{k}$ are involved in computing the attention scores for the words at positions *i* and *j*, the calculation of $W_{q}$ and $W_{k}$ essentially affects the relative positional information between words, resulting in the final position information becoming unpredictable with the training of $W_{q}$ and $W_{k}$.

Nevertheless, programming languages are highly structured and governed by strict logical rules. However, their expressive diversity allows the program logic to be shaped by different coding methods. When writing a program, developers can adopt various coding styles and methodologies based on their requirements and personal preferences. For instance, some programming languages permit multiple syntax rules and idiomatic expressions to convey the same logic, providing developers with the flexibility to choose the most suitable approach for a given context and set of requirements. Therefore, during model training, the ‘RoPE’ positional encoding, which is better suited for processing programming languages, is applied as shown in Equation (6).

(5) RoPE(i,j,k)=R[d,k],

(6) Pos(i)=Embedding(i)+RoPEi.

‘RoPE’ encodes relative positions through absolute position encoding, preserving both positional information and the relationships between relative positions. For the given positions *i* and *j*, with a relative distance d=|i−j|, ‘RoPE’ can be calculated using different methods. Relative positional encoding can be calculated using Equation (5). RoPE(i,j,k) represents the value of the *k*-th dimension of the relative positional encoding between positions *i* and *j*, and R[d,k] represents the value of the *k*-th dimension corresponding to the pair of positions with a distance of ‘d’ in the relative positional encoding matrix. RoPE integrates relative positional encoding with absolute positional encoding to generate a comprehensive positional representation for each position. Specifically, the relative positional encoding is incorporated into the word embeddings, resulting in the final positional encoding, as shown in Equation (6). When computing attention scores, the Transformer network incorporates both absolute and relative positional information. This approach enables it to effectively capture the relative relationships between different positions in a sequence. This enhances performance and efficiency, particularly when processing long sequences in programming languages.

**Figure 7:**
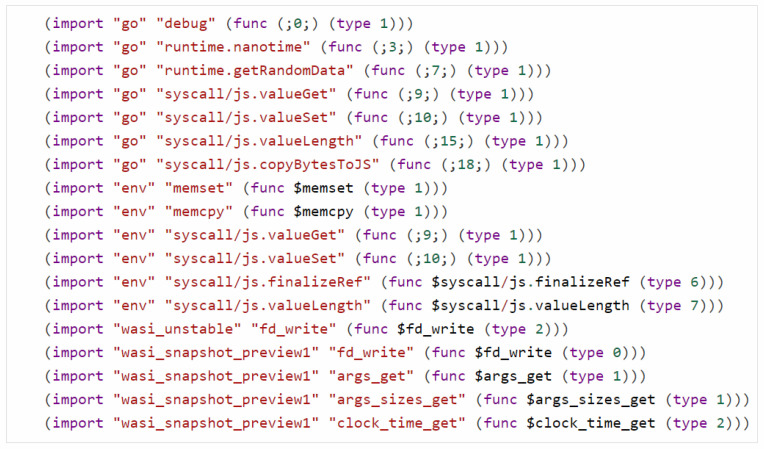
WebAssembly compilation treats all unknown functions as external interfaces, which are listed in the import segment, and must be supported by the symbolic execution engine at runtime.

Symbolic execution engine: Manticore is a symbolic execution tool written in Python, based on SMT2 specification, and supporting Z3, Yices, and CVC4 solvers. So far, Manticore is one of the WebAssembly symbolic execution engines that implements basic symbolic execution of the WebAssembly minimum unit of the operation module. A common WebAssembly program as illustrated in Figure 7. It is clear that different compilations import various external functions, even if these functions have the same name or functionality and belong to the same environment namespaces. Let us take Emscripten as an example. When compiling to WebAssembly programs, ‘syscall/js’ is automatically included, which forces the runtime to either be a browser (Firefox, Chrome, etc), or at least pretend to be one. The APIs in packages such as go, env, wasi_unstable, and wasi_snapshot_preview1 are unknown functions. Therefore, the APIs in the import section of WebAssembly reveal that vulnerability verification has to set up a corresponding runtime environment, otherwise, vulnerability verification cannot be performed.

**Figure 8:**
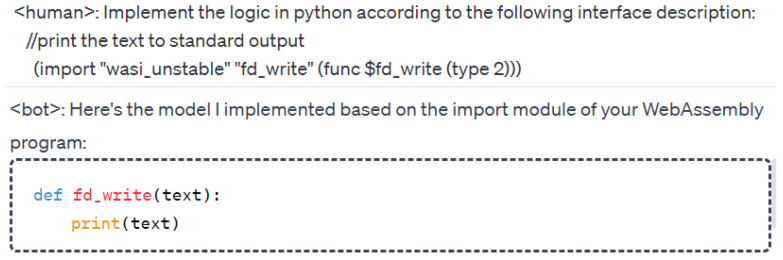
LLM-based automated modeling engine implements modeling in Python 3.x according to the imported function requirements in the WebAssembly program.

When WebAssembly programs are loaded by symbolic execution engines, our approach reads unknown functions from the function import section. Based on the namespace, function names, and contextual function call requirements, our model generates Python code. For instance, the imported function in Figure 8 is well known as a system call, presenting a typical challenge in symbolic execution. Moreover, the symbolic execution engine invokes the abstract function layer based on our Python function instead of the real interface. Our proposed approach is necessary because it allows us to support the verification work of different compilers, which was previously not possible without writing a verification system interface for a specific runtime environment each time.

**Figure 9:**
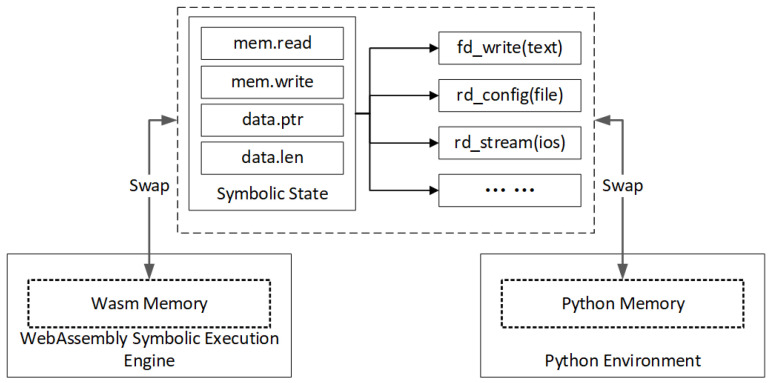
Memory swap between WebAssembly symbolic execution engine and Python environment.

Memory: In our experimental task, our approach must deal with the memory swap problem of symbolic execution. WebAssembly defines a linear, contiguous memory model, allowing WebAssembly modules to utilize a contiguous block of memory. This memory space can be allocated by the host environment and accessed through the memory object of the WebAssembly instance (WebAssembly.Memory). WebAssembly modules can share memory with the host environment. For example, in web browsers, WebAssembly modules can share memory space with JavaScript code, enabling efficient data exchange. This shared memory mechanism is facilitated through the memory object of the WebAssembly instance. Certainly, memory access in WebAssembly is subject to strict bounds and type checking. Memory accesses in WebAssembly modules must be within the linear memory range, and out-of-bound accesses result in runtime errors. This design helps to ensure that the memory accesses in the WebAssembly modules are safe, thereby enhancing the system’s security and stability. The architecture of the symbolic execution engine reveals the internal relationship between the WebAssembly and Python environment as shown in Figure 9.

**Figure 10:**
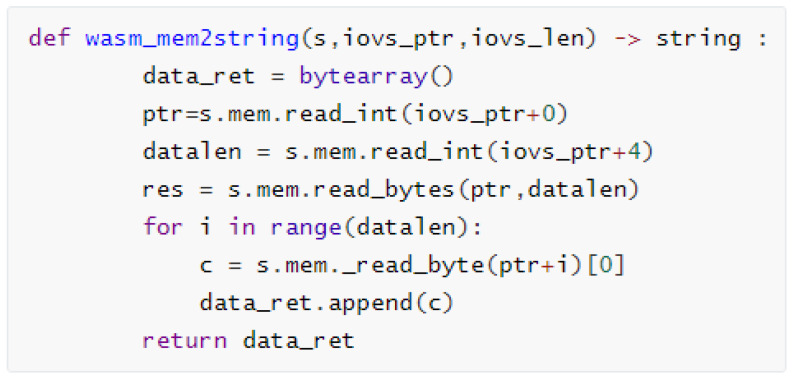
Convert WebAssembly symbolic execution engine memory data to the Python string object via memory pointers and address offsets.

In other words, WebAssembly’s memory isolation prevents external environments, including our Python programs, from directly accessing the memory of an unknown function model. WebAssembly provides the mem-related methods of the symbolic state and then interacts with the outside world. For example, our model implements the ‘wasi_unstable’ function illustrated in Figure 8; the parameter ‘text’ of the ‘fd_write’ function is actually in the ‘mem’ object of the symbolic state. Our approach designs a simple symbolic execution memory swap management between WebAssembly and Python environment, illustrated in Figure 10.

## 5. Experiment and Results

As is well-known, blockchain is a decentralized distributed technology, involving remote procedure call (RPC), distributed features, blockchain frameworks, runtime environments, etc. This is a very challenging vulnerability verification environment for symbolic execution. In order to justify the effectiveness of the LLM-based automated modeling methodology, this section shows that this proposed methodology is applied to smart contracts of the Hyperledger Fabric blockchain, implementing a WebAssembly vulnerability verification automated platform.

**Figure 11:**
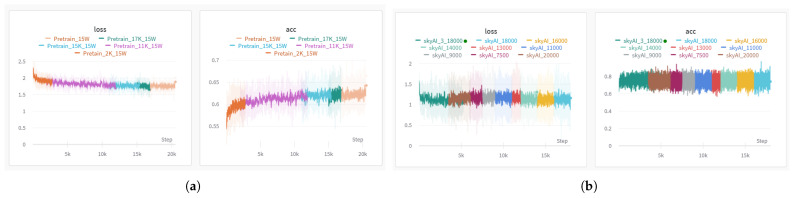
The loss and accuracy of the first stage of training; the pre–training accuracy achieves over 60%. The sub figure (**a**) shows the first stage of pretraining using a unified language learning recovery training method that substantially improves existing language models. The sub figure (**b**) shows The second stage traning involving one sample at a time with left padding.

**Figure 12:**
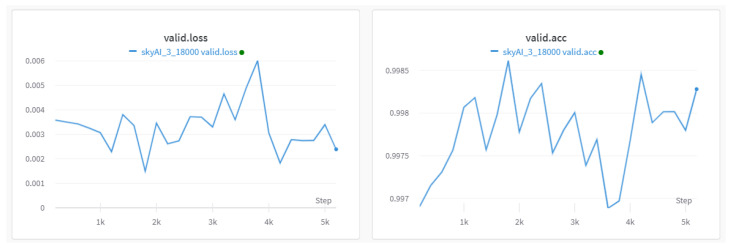
The loss and accuracy of the third stage of training; the valid accuracy achieves 99.8%.

Next, we briefly introduce our model training experiment. The training script is executed on a system with an Nvidia RTX 8000 GPU with 48 GB (NVIDIA, Santa Clara, CA, USA) of memory, a dual Intel E2640 CPU (Intel, Santa Clara, CA, USA), running Ubuntu 22, and equipped with a 7 TB SSD hard disk. The hyperparameter configuration for the model includes a sequence input length of 2048, a dictionary token size of 50,432, a pipeline parallelization size of 9, an embedding size of 6144, a Transformer block layer of 44, a micro-batch size of 18, a gradient accumulation step of 4, and a warm-up step of 128. Our model training involves three stages. For the first stage, we use a unified language learning recovery training method that substantially improves existing language models and their scaling curves with a relatively tiny amount of extra computation, to enhance the generalization of the model. When the training result demonstrated in Figure 11a, the loss value is around 1.78 and the accuracy is 61.92%, the model moves on to the next training stage. The second stage involves training one sample at a time with left padding. The loss calculation for each step employs a full-context approach, allowing the model to gain a comprehensive understanding of semantics. As illustrated in Figure 11b, the model exhibits training accuracy stabilization at approximately 80%. During the final stage of training, we exclusively computed the loss for the response context to enhance the ultimate accuracy of the generated outcomes. Validation accuracy is shown in Figure 12. The whole training process is monitored by the W&B tool; the stages show multiple training sessions with different colors, which are checkpoints saved to test the actual effect of the model.

Ultimately, the trained model is saved in PyTorch(2.6) format with FP16 precision, occupying approximately 44 gigabytes of disk space. We harness a 48-gigabyte GPU card to successfully carry out the inference task, applied in a railway–port–aviation blockchain transportation system. In this system, our verification work is faced with diverse scenarios of unknown functions. The smart contract is developed in Golang, based on the Hyperledger Fabric blockchain framework. The verification of smart contracts requires a mass of unknown function modeling, including a WebAssembly standard interface, a Hyperledger block link interface, a peer-to-peer communication interface, etc. Listing 1 shows a smart contract for a purchase order in railway transportation based on the Hyperledger blockchain.

The security verification of smart contracts is complex, it is due to the decentralized autonomous organization features of blockchain techniques. As shown in the ‘buyOrder’ source code, the rest of the blockchain parts are all interfaces, involving data storage like ‘PutState’, communication protocols like ‘Response’, a framework API like ‘shim.Error’, a data protocol like ‘marshal’, etc. In our previous research, we proposed an optimization methodology of the abstract proxy layer for the smart contract formal verification in the Golang engine framework. In this experiment, our automated modeling addresses the gap in previous research, which required human intervention in the abstract layer code. For instance, the previous security verification work necessitated implementing a ‘JSONProxy’ class in place of the ‘json.Marshal’ method. The abstract layer code programming work was finished by an automated modeling method; the following code in Listing 2 shows the modeling function code in Python.

**Figure 13:**
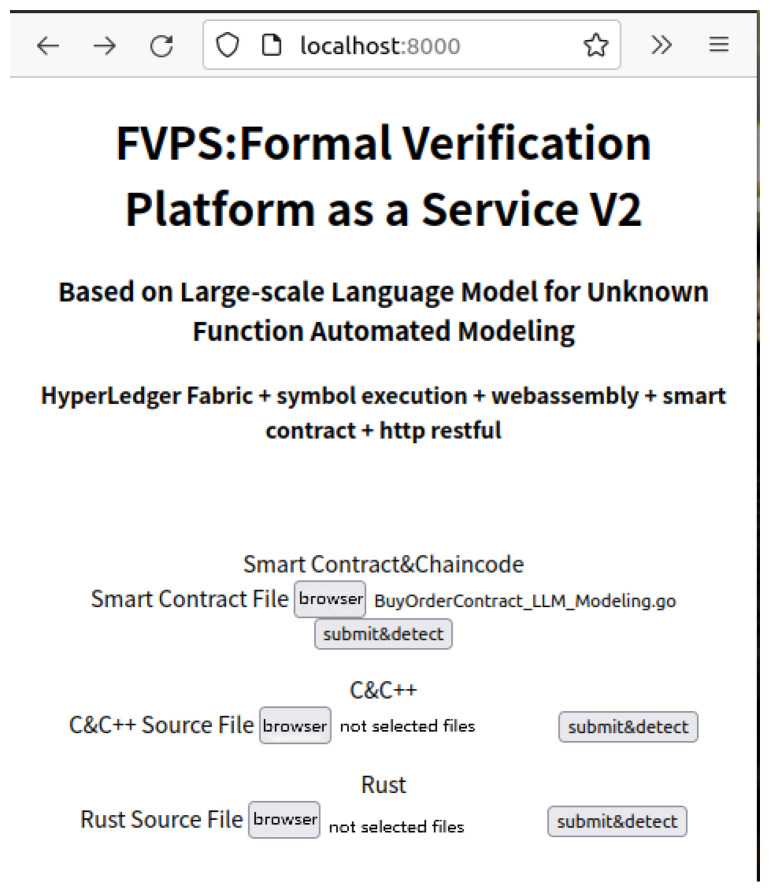
The front-end web page of a formal verification platform for accepting Golang, C/C++, and Rust language programs.

**Figure 14:**
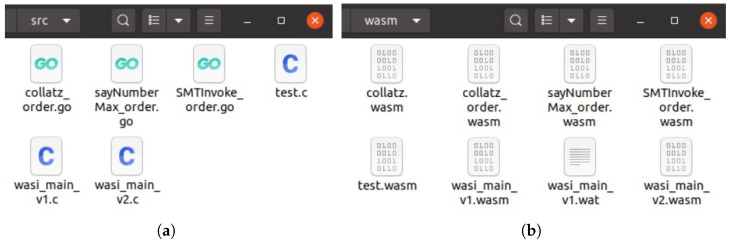
(**a**) The WebAssembly programs are compiled by the formal verification platform from various programming languages; (**b**) the WebAssembly programs are compiled by the formal verification platform from various programming languages.

In addition to this, our formal verification platform based on our automated modeling methodology successfully generates the rest interfaces, such as logging, peer-to-peer response, stub interfaces, etc. To justify the feasibility of our approach, we prepare program source codes in various languages including C/C++, Rust, and Golang; moreover, the user submissions are temporarily stored in the upload directory via the web page illustrated in Figure 13 and Figure 14a.

**Listing 1.** A smart contract of a railway transportation purchase order based on Hyperledger blockchain frameworks.

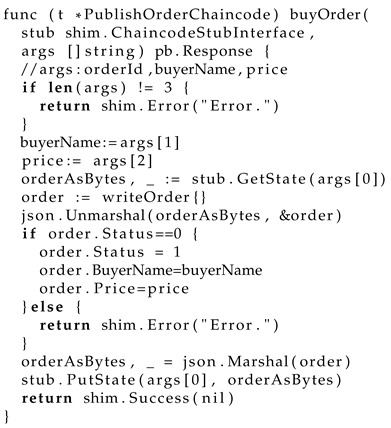



**Listing 2.** The JSON marshal function implementation generated by automated modeling with Python.

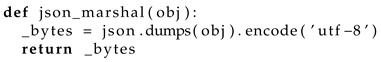



**Figure 15:**
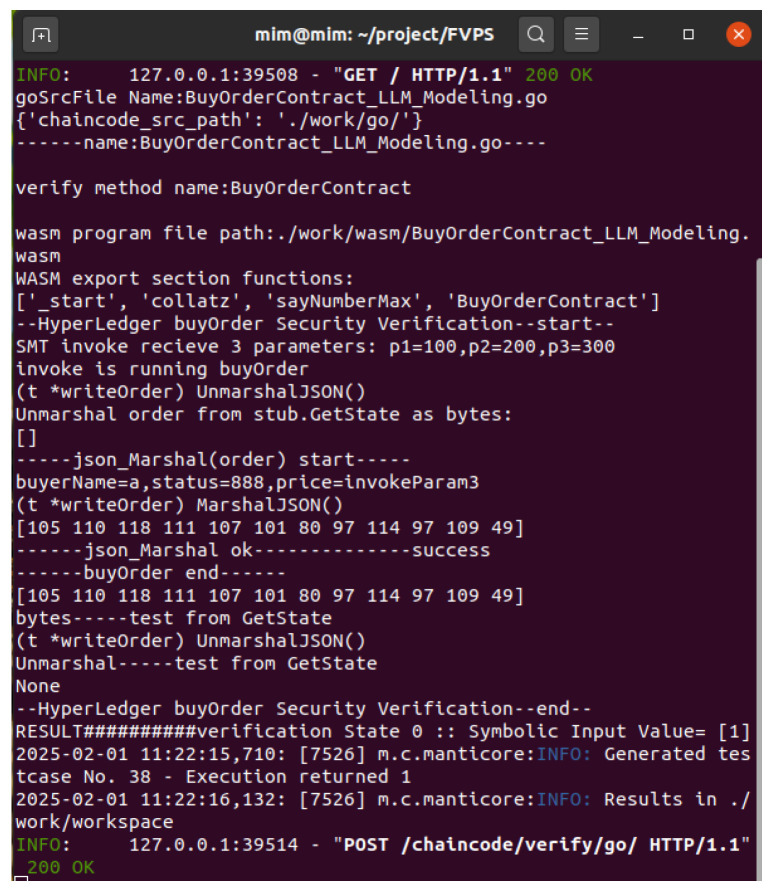
The symbolic execution engine automatically explores a Hyperledger blockchain smart contract, supported by our unknown function automated modeling methodology. The smart contract is explored by symbolic execution, and verification state 0 is successfully generated.

Our previous research compiles them directly into WebAssembly programs, as shown in Figure 14b. Next, the symbolic execution engine loads WebAssembly instructions byte by byte. The import section of the WebAssembly program is recognized by automated modeling based on the LLM, and the corresponding import functions are implemented with Python. Subsequently, the symbolic execution engine starts its security verification explorations. When it encounters unknown functions from the import section of WebAssembly, generative Python function codes like ‘json_marshal’ support upper-level calls. The experimental results in Figure 15 demonstrate the efficiency and automation of our approach.

## 6. Conclusions

Automatic formal verification forms a significant foundation for generic software security. Symbolic execution, as the most important verification technique, is well-known for its automated explorations. However, prior research was always limited by unknown functions from the system, external libraries, and distributed service interfaces in the real world, resulting in industrial application obstruction. Symbolic execution is based on the mixed execution of external functions, and system simulation is an important approach, but it is unwieldy and very expensive to build, for instance, when building an IoT system with numerous sensors. Prior research designed abstract API layers to improve efficiency. But the concrete implementation of the abstract API layer can still take weeks or even months of labor. This paper introduces an innovative approach that leverages Transformer-based large-scale language models to automate the modeling of unknown functions, eliminating the need for manual human intervention. It has great value for improving the automation of symbol execution defect verification. Our experimental findings reveal that challenges previously deemed insurmountable by traditional algorithms and simulation environments can now be effectively addressed through artificial intelligence-driven modeling. Notably, this method significantly enhances the efficiency and adaptability of symbolic execution. For instance, tasks within our prior ’FVPS’ project, which previously required nearly a month’s effort, were completed in mere minutes using this approach. These results underscore the transformative potential of integrating advanced language models into symbolic execution processes, promising substantial improvements in both performance and scalability. Additionally, our experiment justifies multiple-language program verification on WebAssembly, which holds considerable value for WebAssembly applications across various domains, IoT, as well as for traditional generic software security. This work marks an important starting point for the application of LLMs in software security. Challenges remain, such as code generation in diverse environments and ensuring code integrity and accuracy. The coding capabilities of LLMs are also limited when faced with proprietary system APIs, but LLMs have the ability to quickly parse and understand interface documents, quickly generate preliminary code frameworks, and continuously optimize code quality through self-learning in the future, thereby shortening the overall cycle of software security verification. The LLM can also gradually adapt to different demand changes through continuous feedback and updates, forming a complete automated development ecosystem to support diverse system scenarios.

## Figures and Tables

**Table 1:** Function templates of unknown functions in symbolic execution.

user input/output	read data from keyboard/output to screen
OS environment	time/date/register/mac/ip/wifi/cpu
thread	sleep/resume/join/wait
business function	buy/sale/store/purchase/order/etc.
protocol	protobuf/gRPC/JSON/etc.
third libraries	hyperledger/Swing/Android/iOS/QT/etc.
configure file read/write	ini/yml/csv/yaml/xml/json/properties
image file read/write	jpg/png/jpeg/heic/bmp/gif
socket	data read/write from socket/ioctl
http	http request/response
restful api	restful select/create/update/delete interface
web service	simple object access protocol/SOA
websocket	send/recv data from websocket
log system	write log into log file
database CRUD	jdbc/Sqlite/hsql/others
encryption	MD5/DES/AES/SHA/3DES/ECC
data file read/write	jsonl/excel/numpy/csv/xml/dat
audio file read/write	wav/mp3/aac/ogg/aiff
video file read/write	mp4/avi/mkv/mov/wmv/flv
common file read/write	bin/dat/text/binary File

**Table 2:** Training instruction templates, and the categories correspond to three realities. The first involves situations where only interface definitions for functions are available, which may come from WebAssembly’s import functions. The second case occurs if comments for the function can be obtained, such as from the source code. There is also a scenario where the interface documents typically come from third-party vendors. In all three cases, we need to train to enhance the adaptability of unknown function automation modeling.

Templates	Conditions	Unknown Function Modeling Description
A	1. Requirements	According to the function definition and function name, automatically realize the function model.
B	1. Requirements	User requirements make a specific request to generate a function model based on the function definitions.
	2. Declaration	
C	1. Requirements	Richer information produces a more accurate function model;
	2. Comments	it implies more manual intervention, in general,
	3. Declaration	the interface documents are often from third vendors.

## Data Availability

Data are contained within the article.
